# Ileal perineurioma as a cause of intussusception

**DOI:** 10.1590/S1516-31802011000100010

**Published:** 2011-01-06

**Authors:** Sheila Cristina Lordelo Wludarski, Isabel Irene Rama Leal, Herbeth Franco Queiroz, Tulio Marcos Rodrigues da Cunha, Carlos Eduardo Bacchi

**Affiliations:** I MD. Associate pathologist, Pathology Reference Laboratory, Botucatu, São Paulo, Brazil.; II MD. Pathologist, Hospital Regional da Asa Norte (HRAN), Brasília, Federal District, Brazil.; III MD. Urologist, Hospital Regional da Asa Norte (HRAN), Brasília, Federal District, Brazil.; IV MD. General surgeon, Hospital Regional da Asa Norte (HRAN), Brasília, Federal District, Brazil.; V MD, PhD. Chief pathologist, Pathology Reference Laboratory, Botucatu, São Paulo, Brazil.

**Keywords:** Ileum, Immunohistochemistry, Nerve sheath neoplasms, Gastrointestinal tract, Intussusception, Íleo, Imunoistoquímica, Neoplasias da bainha neural, Trato gastrointestinal, Intussuscepção

## Abstract

**CONTEXT:**

Perineuriomas are rare tumors composed of cells resembling those of the normal perineurium. It usually occurs in subcutaneous, soft-tissue or intraneural locations. Very few reports in the literature have described perineuriomas in the gastrointestinal tract, including the stomach, colon and jejunum.

**CASE REPORT:**

We report the clinicopathological and immunohistochemical features of a case of ileal perineurioma that was manifested clinically as intestinal obstruction due to intussusception. Ileal perineurioma has not previously been reported at this anatomical location.

## RESUMO

**CONTEXTO:**

Perineurioma é uma rara neoplasia composta de células que lembram aquelas do perineuro normal e geralmente ocorre no subcutâneo, tecidos moles ou em localização intraneural. Poucos relatos na literatura descrevem perineuriomas no trato gastrointestinal incluindo estômago, cólon e jejuno.

**RELATO DE CASO:**

Os autores apresentam as características clinicopatológicas e imunoistoquímicas de um caso de perineurioma ileal apresentandose clinicamente por obstrução intestinal decorrente de intussuscepção. Perineurioma ileal não havia sido descrito até o momento nessa localização anatômica.

## INTRODUCTION

Perineuriomas are uncommon benign neoplasms that occur mainly in the subcutis, but also arise in soft tissues or intraneural locations. The neoplastic cells comprising perineuriomas resemble those of the normal perineurium. These neoplasms were first described by Lazarus and Trombetta in 1978.^1^ An unusual variant of soft-tissue perineurioma has been characterized by Fetsch and Miettinen as a sclerosing variant.^2^ Perineuriomas have also been reported in unusual locations such as the kidneys^3^ and the paratesticular region.^4^ Very few reports^5-10^ have described perineuriomas in the gastrointestinal tract, and these have mainly been in the stomach, colon and jejunum. We report the clinicopathological and immunohistochemical features of a case of ileal perineurioma, of soft-tissue type, manifested clinically through intestinal obstruction due to intussusception. To the best of our knowledge, perineurioma has not been previously reported in this anatomical location.

## CASE REPORT

A previously healthy 25-year-old white male presented with complaints of abdominal pain for two weeks. The pain was mainly located in the periumbilical area and was associated with nausea. There was no fever. The pain progressively increased and the patient started presenting episodes of vomiting. Abdominal radiographs and ultrasound scans revealed findings of intussusception. Laparotomy was performed and ileal intussusception was found 60 cm from the ileal-cecal valve, caused by a 5-cm tumor involving the intestinal wall of the ileum. The tumor was surgically removed and the patient's postoperative evolution was uneventful.

Clinical information was obtained from the patient's records. The gross pathological examination revealed an ulcerated polypoid tumor of the ileum measuring 5 cm across the greatest diameter. The cut surface showed a bright whitish tumor mass with areas of hemorrhage.

**Figure 1.**
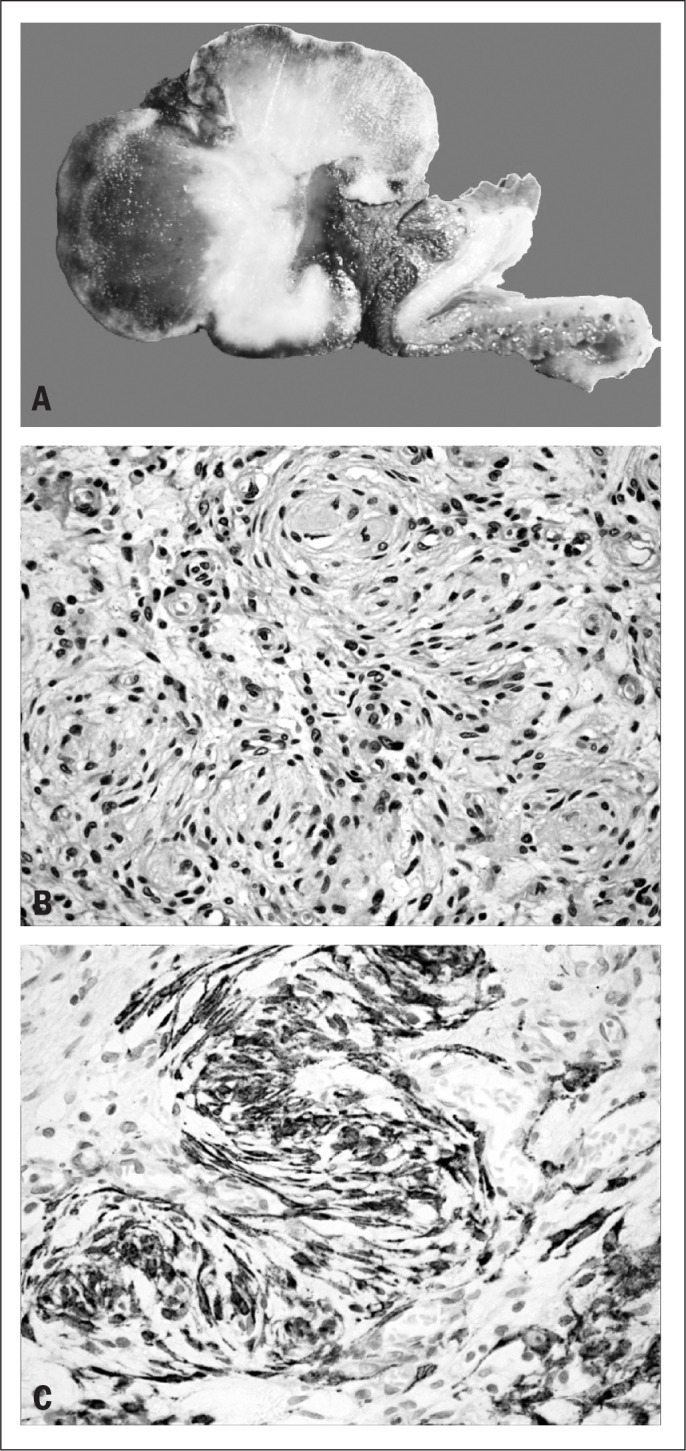
**A:** Macroscopic findings from ileal perineurioma. **B:** Hematoxylin and eosin. Proliferation of bland spindle cells with ovoid to elongated nuclei and indistinct cytoplasm. Note the tendency for the tumor cells to be located around vessels in whorls of striking appearance. **C:** Immunohistochemical expression of epithelial membrane antigen (EMA) by the neoplastic cells.

The diagnosis was based on examination of histological sections stained with hematoxylin and eosin. Microscopic examination revealed a neoplasm involving the submucosa and the muscle layer of the ileum. The tumor was composed of bland spindle cells with ovoid to elongated nuclei and indistinct cytoplasm. It was highly vascularized, with the presence of many small rounded vessels. There was a tendency for the tumor cells to be located around these vessels in whorls of striking appearance (**Figure 1A**). No clear-cut atypia or pleomorphism was seen. In some areas, the tumor seemed to infiltrate the muscle layer focally. The stroma was hyalinized in some areas and myxoid in others.

The expression of epithelial membrane antigen (EMA), claudin-1, S-100 protein, CD117, CD34, cytokeratin and smooth-muscle actin and desmin was investigated by means of immunohistochemistry using a standard avidin-biotin method. Immunohistochemical analysis revealed diffuse expression of EMA (**Figure 1B**) and claudin-1 by the neoplastic cells, but the cells were negative for S-100 protein, CD117, CD34, cytokeratin, smooth muscle actin and desmin.

## DISCUSSION

Perineuriomas are rare benign peripheral nerve sheath tumors arising mainly in the subcutis, but they have also been described in soft tissue and intraneural locations.^11^ They occur in adults and are not associated with neurofibromatosis types 1 and 2. The most common morphological finding from soft-tissue perineuriomas is the presence of neoplastic spindle-cell populations composed of slender fibroblastic-like cells arranged in a vague fascicular, storiform or whorl-forming pattern. Perineuriomas are very rare outside of the subcutis, soft tissue and intraneural locations.

They have been reported in the kidneys^3^ and the paratesticular region.^4^ Very few reports of perineuriomas arising in the gastrointestinal tract have been published.^5-10^ Hornick et al.^5^ first described perineuriomas of the intestine. They presented 10 cases of intestinal perineuriomas, of which nine were located in the colon and one in the jejunum, with no cases located in the ileum. Additional reports of gastrointestinal perineuriomas have included rare cases of perineurioma of the stomach,^6-8^ a case of perineurioma of the esophagus^9^ and a case of benign hybrid perineurioma-schwannoma of the colon.^10^ In the gastrointestinal tract, perineuriomas usually present as asymptomatic intramucosal lesions, which are incidentally detected by screening tests. Here, as shown in **Table 1**, we described the first case of ileal perineurioma of a patient who presented with signs and symptoms relating to intussusception. Most of the morphological features seen in this case were also observed by Hornick et al.,^5^ including the presence of uniform bland spindle cells with ovoid to elongated nuclei and pale indistinct eosinophilic cytoplasm, myxoid or collagenous stroma, and rare mitotic figures. The result from immunophenotyping our case consisted of findings that typically occur in perineuriomas, i.e. strong expression of EMA and claudin-1 with no expression of S-100 protein. It has been stated that virtually all perineuriomas express EMA,^12,13^ while they are also positive for claudin-1 in about 90% of the cases. Both of these markers reveal membranous patterns through immunostaining. Moreover, perineuriomas have been found to be negative for S-100 protein.

**Table 1.** Literature review* on ileal perineurioma

Electronic databases	Search strategies	Results	
		Found	Related
PubMed	Perineurioma AND Ileum Limits: Case Reports	63	0
SciELO	Perineurioma	4	0
Lilacs	Perineurioma	23	0
Scirus	Perineurioma AND Ileum	15	0

* Performed on August 20, 2010.

The most important differential diagnoses in this clinical pathological context (tumors involving the intestinal wall) are with gastrointestinal stromal tumor (GIST) and schwannoma.^6,7^ GISTs are usually composed of bland spindle and/or epithelioid cells without the uniquely perivascular location of the tumor cells that was seen in the present case, and over 95% of GISTs are positive for CD117 (KIT), while often negative for EMA. Schwannomas are neoplasms that rarely occur in the gastrointestinal tract, with the exception of the stomach. In these rare cases located in the gastrointestinal tract, they show distinct morphological findings including highly cellular areas, presence of thick wall vessels, inflammatory infiltrate and sometimes germinal center formation. Immunohistochemical analysis can easily separate schwannomas from perineuriomas, since the former are positive for S-100 protein and glial fibrillary acidic protein and the latter are positive for EMA. Other differential diagnoses in such cases that are worth mentioning are with leiomyomas and leiomyosarcomas. These tumors have a more fascicular pattern of growth with no tendency for the tumor cells to be located around small vessels. Leiomyomas and leiomyosarcomas characteristically demonstrate expression of muscle markers such as desmin and smooth muscle actin, and our case was negative for both of these markers.

According to Hornick et al.,^5^ it seems that perineuriomas of the gastrointestinal tract probably have a benign clinical course. This case was very challenging to diagnose, because it was a rare tumor in an unusual location.
